# Idiopathic Ventricular Arrhythmias Ablated in Different Subregions of the Aortic Sinuses of Valsalva: Anatomical Distribution, Precordial Electrocardiographic Notch Patterns, and Bipolar Electrographic Characteristics

**DOI:** 10.3389/fcvm.2021.778866

**Published:** 2021-12-20

**Authors:** Sixian Weng, Zhengqin Zhai, Min Tang, Bin Zhou, Lei Ding, Fengyuan Yu, Yingjie Qi, Hongda Zhang, Tianjie Feng, Shu Zhang

**Affiliations:** ^1^State Key Laboratory of Cardiovascular Disease, Department of Cardiology, Cardiovascular Institute, Fuwai Hospital, National Center for Cardiovascular Diseases, Chinese Academy of Medical Sciences and Peking Union Medical College, Beijing, China; ^2^China-Japan Friendship Hospital, Beijing, China

**Keywords:** ventricular arrhythmias, aortic sinuses of Valsalva, bipolar electrogram, target distribution, precordial notch, radiofrequency catheter ablation

## Abstract

**Background:** Little is known about the differences among ventricular arrhythmias (VAs) ablated in different subregions of the aortic sinuses of Valsalva (ASVs). We aim to investigate the distribution, precordial electrocardiographic patterns, and bipolar electrogram characteristics of VAs ablated in different subregions of the ASVs.

**Methods:** We divided the right ASV and the left ASV into a total of 6 subregions and studied 51 idiopathic VAs ablated first time successfully in the ASVs.

**Results:** These 51 VAs were inhomogeneously distributed among the 6 subregions, which comprised the right-lateral ASV (1/51), the right-anterior ASV (11/51), the regions along the right (13/51) and left (9/51) sides of the ASV junction, the left-anterior ASV (5/51), and the left-lateral ASV (12/51). Fractionated potentials were dominant (39/51, 76%) among the 3 types of target electrograms. From the right-lateral ASV to the left-lateral ASV, the percentage of fractionated potentials gradually decreased from 100 to 59%. A precordial rebound notch in V3-V4 or V4-V5 had sensitivity of 90.9%, specificity of 85.0%, and negative predictive value (NPV) of 97.1% to predict VAs ablated in the right-anterior ASV. A precordial rebound notch in V2-V3 had sensitivity of 50.0%, specificity of 94.9%, and NPV of 86.0% to predict VAs ablated in the left-lateral ASV.

**Conclusion:** VA targets were mainly distributed in the anterior and the left-lateral ASVs. Fractionated potentials were common among target electrograms, especially in theright-anterolateral ASV. Precordial electrocardiographic rebound notch has high predictive accuracy in identifying different subregions of the ASVs as target ablation sites.

## Introduction

Idiopathic ventricular arrhythmias (VAs) with the electrocardiographic inferior axis frequently originate from the aortic sinuses of Valsalva (ASVs) (1, 2). Compared to VAs originating from the right ventricular outflow tract (RVOT), mapping and ablation are usually challenging for VAs from the ASV (ASV-VAs) due to the complicated anatomical construction of the ASVs, confusion of ECG locations, and the difficulty of confirming target electrograms. Thus, a lower radiofrequency catheter ablation (RFCA) success rate was observed for ASV-VAs than for VAs originating from the RVOT. Previous studies have described the electrocardiographic or electrophysiological characteristics of VAs from the left ASV (3), the right ASV (4), and the left-right commissure (5, 6), while little is known about the distributions of VAs in the subregions of the ASVs. In this study, we further divided the left and right ASVs into 6 subregions, with the aim of investigating the target distribution, the specific precordial electrocardiographic patterns, and the bipolar electrographic characteristics of VAs ablated in different subregions.

## Methods

### Study Population

This single-center retrospective study enrolled 65 consecutive patients with inferior axis deviation and monomorphic VAs who had no RFCA history and were referred for a first acute successful RFCA in the ASVs from September 2016 to September 2020 in the Department of Cardiology, Fuwai Hospital. The exclusion criteria were as follows: (1) structural heart disease was diagnosed before RFCA and (2) VAs with the same ECG morphology that had been ablated in the 1st procedure recurred during a follow-period of at least 6 months. The clinical data that were analyzed in the study included baseline characteristics, standard 12-lead ECGs, intracardiac electrograms, 3-dimensional electroanatomic mapping data, imaging information, and follow-up outcomes. This study was approved by the Ethics Committee of Fuwai Hospital (approval no. 2021-1523), Chinese Academy of Medical Sciences, and followed the Declaration of Helsinki. A written informed consent was obtained from all the patients.

### Three-Dimensional Mapping and RFCA

After discontinuing antiarrhythmic drugs for at least 5 half-lives and completing preoperative examinations, patients underwent mapping and RFCA procedures. We obtained the 12-lead ECGs (bandpass filter: 0.05–100 Hz) and intracardiac electrograms (bandpass filter: bipolar 30–500 Hz; unipolar 0.1–500 Hz) from a multichannel electrophysiology recorder (Bard Electrophysiology, Lowell, Massachusetts, USA). Activation mapping was performed using a 3-dimensional electroanatomic mapping system (CARTO3, Biosense Webster Incorporation, Diamond Bar, California, USA) with an irrigated catheter (NaviStar ThermoCool, Biosense Webster Incorporation, Diamond Bar, California, USA) or contact force catheter (Thermocool SmartTouch, Biosense Webster Incorporation, Diamond Bar, California, USA). The mapping catheter was advanced via an SL1 or SR0 long sheath, if necessary. Heparin was administered after accessing the femoral artery and was used to maintain an activated clotting time of 200–250 s. For patients with a transitional zone index (7) ≥ −0.5, we performed 3-dimensional mapping in the RVOT first; those with a transitional zone index < −0.5 underwent direct mapping in the ASVs by a transaortic approach. Electroanatomic maps were constructed via fast anatomic mapping, point-by-point mapping, or intracardiac echocardiography (ICE) reconstruction (Soundstar, Biosense Webster Incorporation, Diamond Bar, California, USA). An ideal target for ablation was based on 3 criteria: (1) the earliest activated site during mapping (≥20 ms earlier than the QRS onset of VAs); (2) a synchronized onset of distal bipolar and unipolar (QS shape) electrogram; and (3) if possible, a pace mapping (match ≥ 95%) was performed to confirm the final target if the earliest activated site was unclear or showed little difference (<5 ms) from other sites. Before ablation, we used selective coronary angiography (using a pigtail catheter or irrigated catheter) or an ICE image to confirm that the distance between the target and the coronary ostia was greater than 5 mm. RFCA was delivered with a maximum power of 35 W and a maximum temperature of 55°C (irrigated flow rate: 2 ml/min) for up to 60–90 s. An effective RFCA was defined as reduction, disappearance, or acceleration with the same ECG morphology of VAs within the 1st 10 s. If RFCA did not influence VAs, it was discontinued and the catheter was repositioned. The endpoint for RFCA consisted of the disappearance of VAs for at least 20 min and the non-inducibility of VAs during isoproterenol infusion and/or programmed stimulation.

### Subregions of the ASV

**Figure 1:**
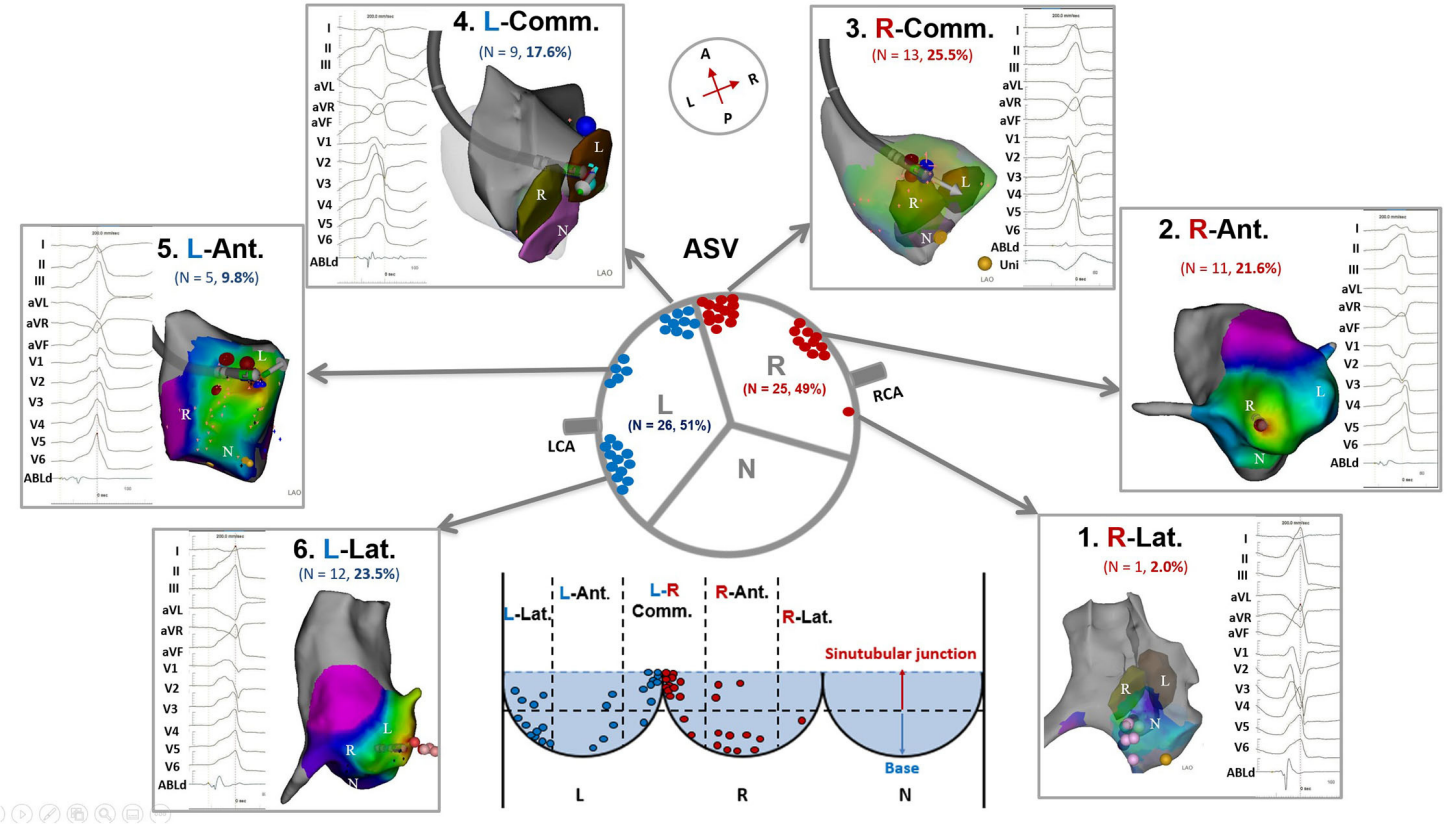
Anatomic distributions of ventricular arrhythmias ablated in the ASVs. ASV, aortic sinus of Valsalva; N, non-coronary ASV; L, left ASV; R, right ASV; RCA, right coronary artery; LCA, left coronary artery; R-Lat., right-lateral ASV; R-Ant., right-anterior ASV; R-Comm., right side adjacent to the left-right commissure; L-Comm., left side adjacent to the left-right commissure; L-Ant., left-anterior ASV; L-Lat., left-lateral ASV.

To determine the differences of VAs in electrocardiographic/electrophysiological characteristics from different regions in the ASVs, we further divided the right and left ASVs into 6 subregions (Figure 1): (1) Right-lateral ASV: the lateral and posterior portions of the right ASV, usually located behind the right coronary ostium; (2) Right-anterior ASV: the anterior portion (center section) of the right ASV, which contained the nadir of the semilunar hinges, usually located between the right coronary ostium and the adjacent left-right commissure; (3) Right side adjacent to the left-right commissure; (4) Left side adjacent to the left-right commissure; (5) Left-anterior ASV: the anterior portion (center section) of the left ASV, which contained the nadir of the semilunar hinges, usually located between the left coronary ostium and the adjacent left-right commissure; and (6) Left-lateral ASV: the lateral and posterior portions of the left ASV, usually located behind the left coronary ostium. The location of different subregions was based on 3-dimensional maps and imaging confirmation (ICE and/or angiography).

### Analysis of ECG and Intracardiac Electrograms

**Figure 2:**
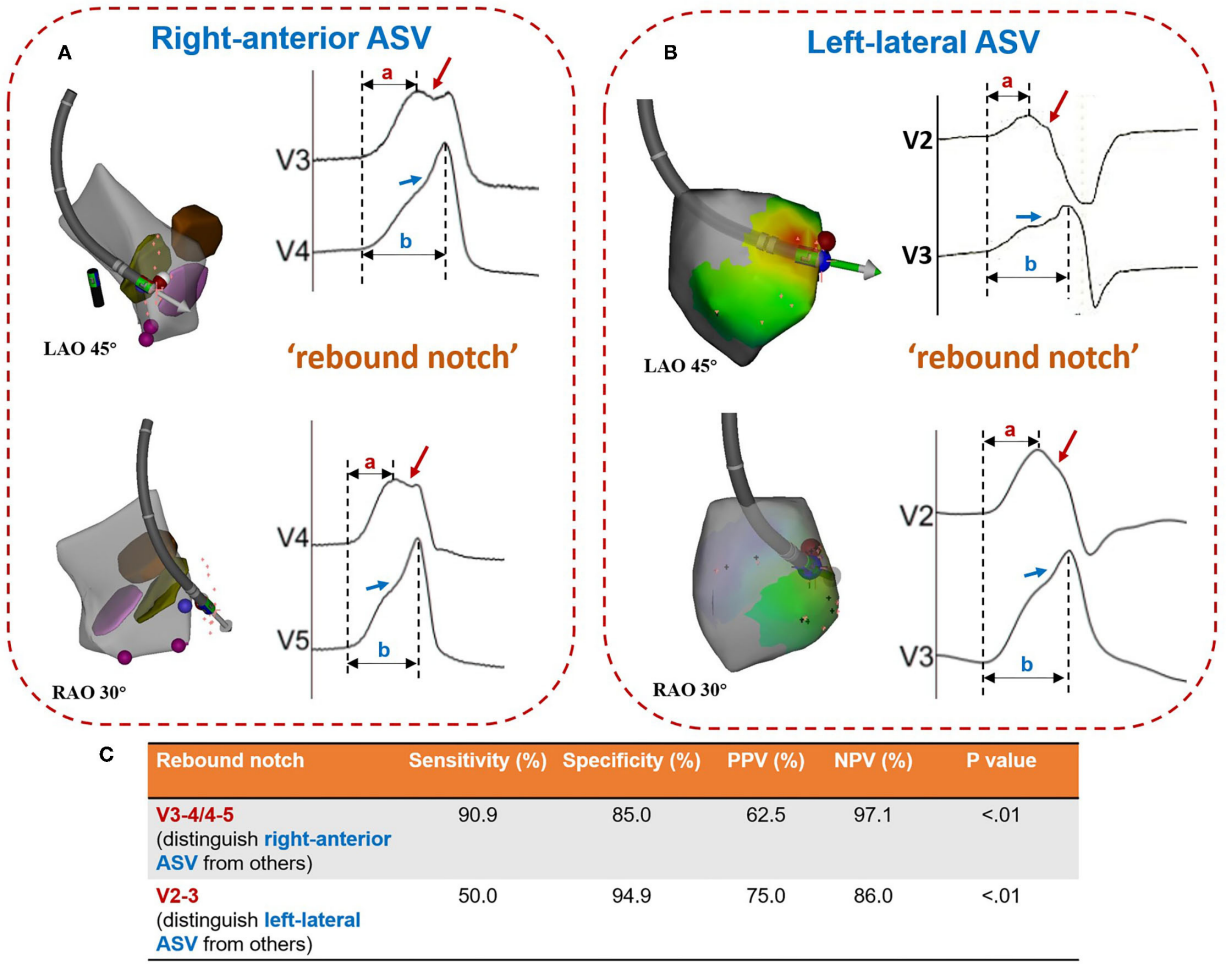
Patterns of rebound notches on the precordial lead. **(A)** Left-sided (V3-V5) rebound notches (a downstroke notch on the earlier lead followed by an upstroke notch on a later lead; peak R-wave deflection interval: (a < b) is more common in ventricular arrhythmias ablated in the right-anterior ASV. **(B)** Right-sided (V2-V3) rebound notches are more common in ventricular arrhythmias ablated in the left-lateral ASV. **(C)** The accuracy of rebound notches in identifying ventricular arrhythmias originating from the right-anterior ASV and/or the left-lateral ASV. ASV, aortic sinus of Valsalva; PPV, positive predictive value; NPV, negative predictive value.

**Figure 3:**
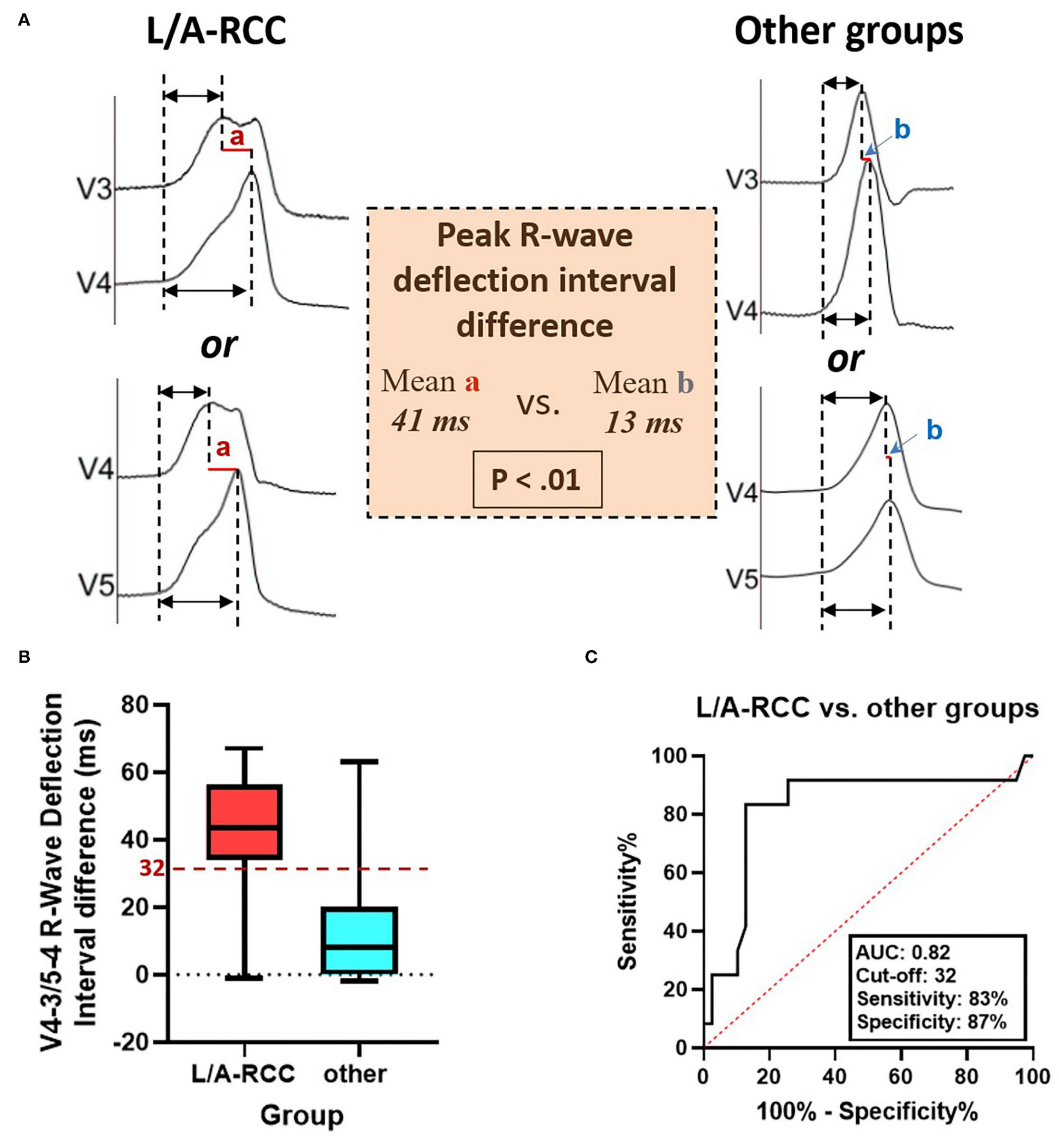
The peak R-wave deflection interval difference between V4-V3 and V5-V4 during ventricular arrhythmias ablated in the ASVs. **(A)** The R-Ant./Lat. group had a longer peak R-wave deflection interval difference between V4-V3 and V5-V4 than other groups. **(B)** The box-and-whisker plots show the V4-V3 or V5-V4 peak R-wave deflection interval difference between R-Ant./Lat. and other groups. **(C)** The receiver operating characteristic analysis for peak R-wave deflection interval difference between V4-V3 and V5-V4 in patients with ventricular arrhythmias ablated in the ASVs. ASV, aortic sinus of Valsalva; R-Ant./Lat., right-lateral/anterior ASV; AUC, area under the curve.

**Figure 4:**
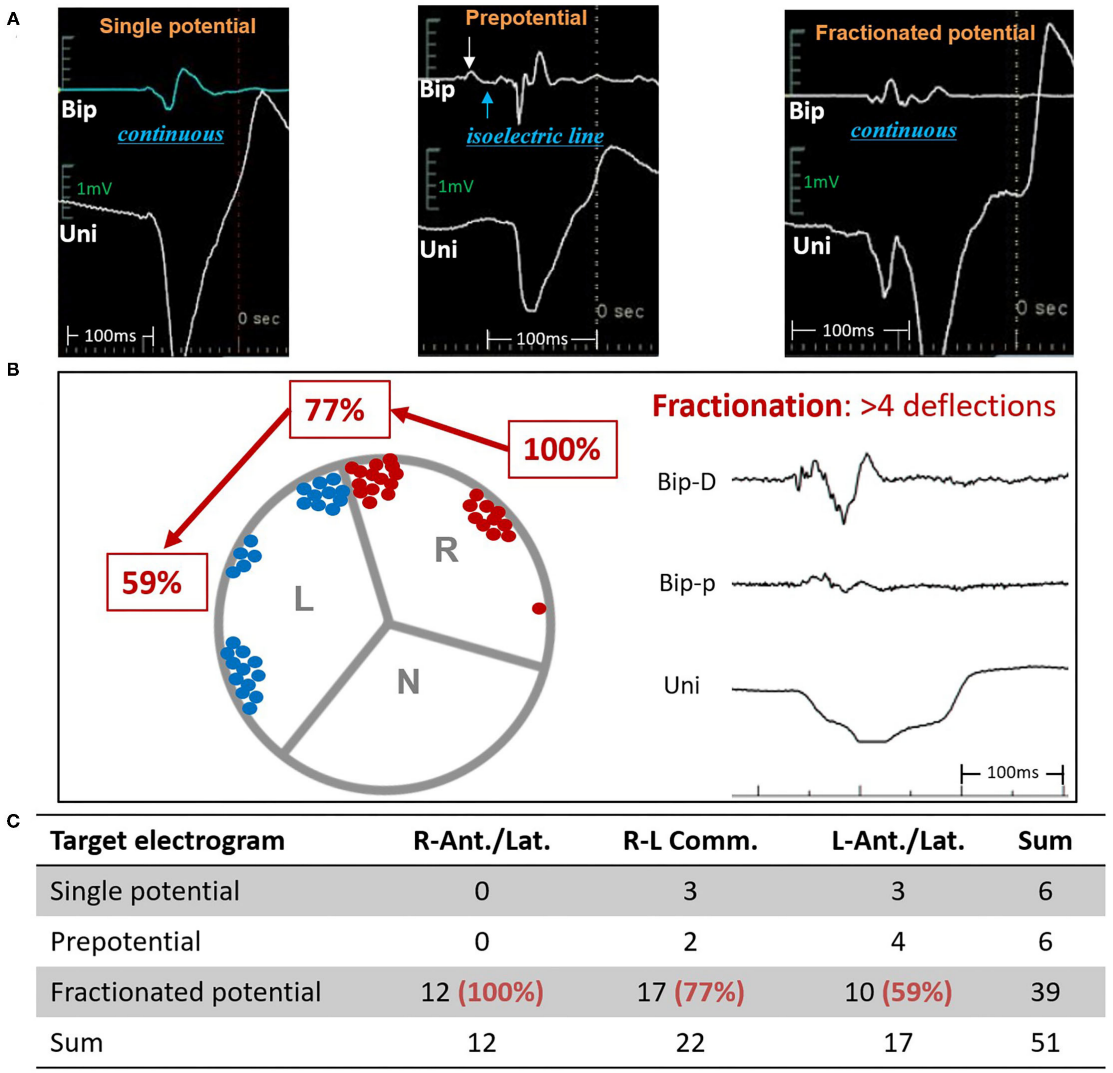
Distributions and characteristics of the target bipolar electrogram. **(A)** 3 types of target electrograms. **(B)** Distributions and **(C)** proportions of fractionated potentials. Bip, bipolar electrogram; Uni, unipolar electrogram; ASV, aortic sinus of Valsalva; N, non-coronary ASV; L, left ASV; R, right ASV; R-Ant./Lat., right-lateral/anterior ASV; R-L Comm., left-right commissure; L-Ant./Lat., left-lateral/anterior ASV.

A notch was defined as an extra deflection (not in initiation and termination) in downstroke or upstroke. A precordial electrocardiographic rebound notch of VAs was defined as a downstroke notch on the earlier lead followed by an upstroke notch on a later lead (Figure 2); this feature can also be quantified as the difference of peak R-wave deflection interval (from the earliest QRS onset to the peak R-wave deflection) (8) between the adjacent precordial leads (Figure 3). Data from the bipolar electrograms of the target in different ASV subregions were focused on amplitude, duration, and earliest activation. The bipolar electrogram characteristics of the target were reflected in the deflection and potential elements (Figure 4).

### Follow-Up

All the patients underwent a 12-lead ECG and 24-h Holter monitoring on the same day, 1 month, 3 months, 6 months, and 12 months after the procedure. Meanwhile, outpatient or telehealth visits were performed when patients experienced clinical symptoms such as palpitation. Recurrence was defined as a clinical VA (same ECG morphology) burden ≥ 2% in 24-h Holter monitoring.

### Statistical Analysis

Continuous variables are expressed as the mean ± SD (normal distribution) or median with interquartile range (non-normal distribution), while categorical variables are described as numbers or percentages. Continuous variables were compared using the one-way ANOVA (normal distribution) or the Mann–Whitney *U* test (abnormal distribution). Categorical variables were compared using the chi-squared test. The Bonferroni correction was applied to multiple comparisons among the different subregions. A box-and-whisker graph and the receiver operating characteristic curve were used to obtain the best diagnostic values. A two-sided *p*-value < 0.05 was considered as statistically significant. Data analysis was performed using the SPSS version 21 (IBM Corporation, Armonk, New York, USA). Graphs were obtained using the Prism version 8.0 (GraphPad Software Incorporation, La Jolla, California, USA).

## Results

### Characteristics of the Patient

After excluding 14 patients (4 patients with structural heart disease and 10 patients with VA recurrence), a total of 51 patients with successfully ablated VAs in the ASVs were enrolled in this study. More than half of the patients were male. Most of them were middle aged (Q1–Q3, 39–59 years). All the patients presented with normal cardiac structure and function with the clinical symptoms of palpitation and/or presyncope before ablation. The details of the baseline characteristics are shown in Table 1.

**Table 1:** Baseline characteristics.

Patient characteristics (*N* = 51)	
Age, y	53 (39–59)
Male, n (%)	31 (61)
Clinical symptoms, n (%)	
Palpitations	51 (100)
Presyncope	1 (2)
Syncope	0
Clinical VAs, n (%)	
Only PVCs	41 (80)
PVCs, non-sustained VT	8 (16)
PVCs, sustained VT	2 (4)
LVEDD (mm) before ablation	49 (45–54)
LVEF (%) before ablation	64 (60–65)
No. of patients LVEF <50%	0
VA burden before ablation, beats/24 h	24445 (15000–36000)

*Values are presented as the median (Q1–Q3) or n (%). VA, ventricular arrhythmia; PVC, premature ventricular contraction; LVEDD, left ventricular end-diastolic dimension; LVEF, left ventricular ejection fraction*.

### Characteristics of Distribution and Bipolar Electrograms in Ablation Target

A total of 20 patients underwent remapping and reablation in the ASVs after failed attempts to find an ideal target and/or achieved an effective RFCA (reduction, elimination, and morphology change of VAs) in the RVOT. All the VAs were eventually mapped and ablated successfully in the ASVs. Targets were most commonly (39/51, 76%) located in the anterior portion (in front of the coronary ostia) of the ASVs, which comprised the right-anterior ASV (11/51, 21.6%), the right side adjacent to the left-right commissure (13/51, 25.5%), the left side adjacent to the left-right commissure (9/51, 17.6%), and the left-anterior ASV (5/51, 9.8%). Only 1 VA was located in the right-lateral ASV, while 12 VAs (12/51, 23.5%) were located in the left-lateral ASV (Figure 1). The mean earliest activation on bipolar electrogram was 28 ms. As shown in Table 2, when compared to VAs in the left-right commissure, those in the left-anterior/lateral ASVs had lower amplitudes of the target bipolar electrogram (0.50–1.16 vs. 0.35–0.67, *p* = 0.044). A total of 3 kinds of target bipolar electrograms were found in this study including the single potential (6/51, 12%), the prepotential (6/51, 12%), and the fractionated potential (39/51, 76%). From the right-lateral ASV to the left-lateral ASV, the percentage of fractionated potential gradually decreased from 100 to 59% (Figure 4 and Supplementary Table 1).

**Table 2:** Bipolar electrogram and ECG characteristics during ventricular arrhythmias.

	**R-Ant./Lat. (*N* = 12)**	**R-L comm. (*N* = 22)**	**L-Ant./Lat. (*N* = 17)**	***P* value**
Target amplitude, mV	0.56 (0.33–0.99)^a, b^	0.76 (0.50–1.16)^a^	0.41 (0.35–0.67)^b^	0.044*
Target duration, ms	138.9 ± 14.5	139.1 ± 42.6	124.9 ± 35.4	0.416
Earliest activation, ms	25.8 ± 6.0	27.4 ± 7.4	29.5 ± 8.3	0.469
Lead I morphology, *n* (%)				
R/RR'/RSR'	11 (91.7)^a^	9 (40.9)^b^	2 (11.8)^b^	<0.05^*^
Rs (R/S ≥ 1)	0	6 (27.3)	3 (17.6)	>0.05
rS (R/S <1)	0	4 (18.2)	3 (17.6)	>0.05
QS, QRS	1 (8.3)^a^	3 (13.6)^a^	9 (52.9)^b^	<0.05^*^
Precordial transition, *n* (%)				
V1	1 (8.3)^a^	7 (31.8)^a, b^	9 (52.9)^b^	<0.05^*^
V1-2	1 (8.3)	3 (13.6)	1 (5.9)	>0.05
V2	1 (8.3)	2 (9.1)	0	>0.05
V2-3	4 (33.3)	5 (22.7)	7 (41.2)	>0.05
≥V3	5 (41.7) ^a^	5 (22.7) ^a, b^	0^b^	<0.05^*^
Precordial rebound notch, *n* (%)				
None	0^a^	16 (72.7)^b^	10 (58.8)^b^	<0.05^*^
V1-2	1 (8.3)	0	0	>0.05
V2-3	0	2 (9.1)	6 (35.3)	>0.05
V3-4	9 (75) ^a^	2 (9.1) ^b^	1 (5.9) ^b^	<0.05^*^
V4-5	2 (16.7)	2 (9.1)	0	>0.05

*Values are presented as the mean ± SD, median (Q1–Q3), or n (%). ASV, aortic sinus of Valsalva; R-Ant./Lat., right-lateral/anterior ASV; R-L Comm., left-right commissure; L-Ant./Lat., left-lateral/anterior ASV. ^a or b^Different letters indicate intergroup significance; e.g., for the “target amplitude,” significance is shown between R-L Comm. and L-Ant./Lat*.

*The Bold values and the symbol*

* * highlight the P value < 0.05*.

### Electrocardiogram Characteristics of VAs Originating From Different Subregions of the ASV

A positive “R” wave in the initiation and termination of lead I was more common (91.7 vs. 40.9 vs. 11.8%, *p* < 0.05) in VAs from the right-anterior/lateral ASV than in those from other subregions. From the right-lateral ASV to the left-lateral ASV, the percentage of a negative “S/QS” in lead I increased gradually (8.3 vs. 13.6 vs. 52.9%, *p* < 0.05). More than 80% of VAs from the right-anterior/lateral ASV precordial transited ≥ V2. A precordial transition before V2 was most common in the left-right commissure and the left-anterior/lateral ASV (45.4 vs. 58.8%). A precordial electrocardiographic rebound notch was more common in VAs from the right-anterior/lateral ASV (100 vs. 28.3 vs. 41.2%) than in those from the left-right commissure or the left-anterior/lateral ASV. The detailed ECG comparisons are shown in Table 2.

### Diagnostic Value of Precordial Rebound Notch

**Figure 5:**
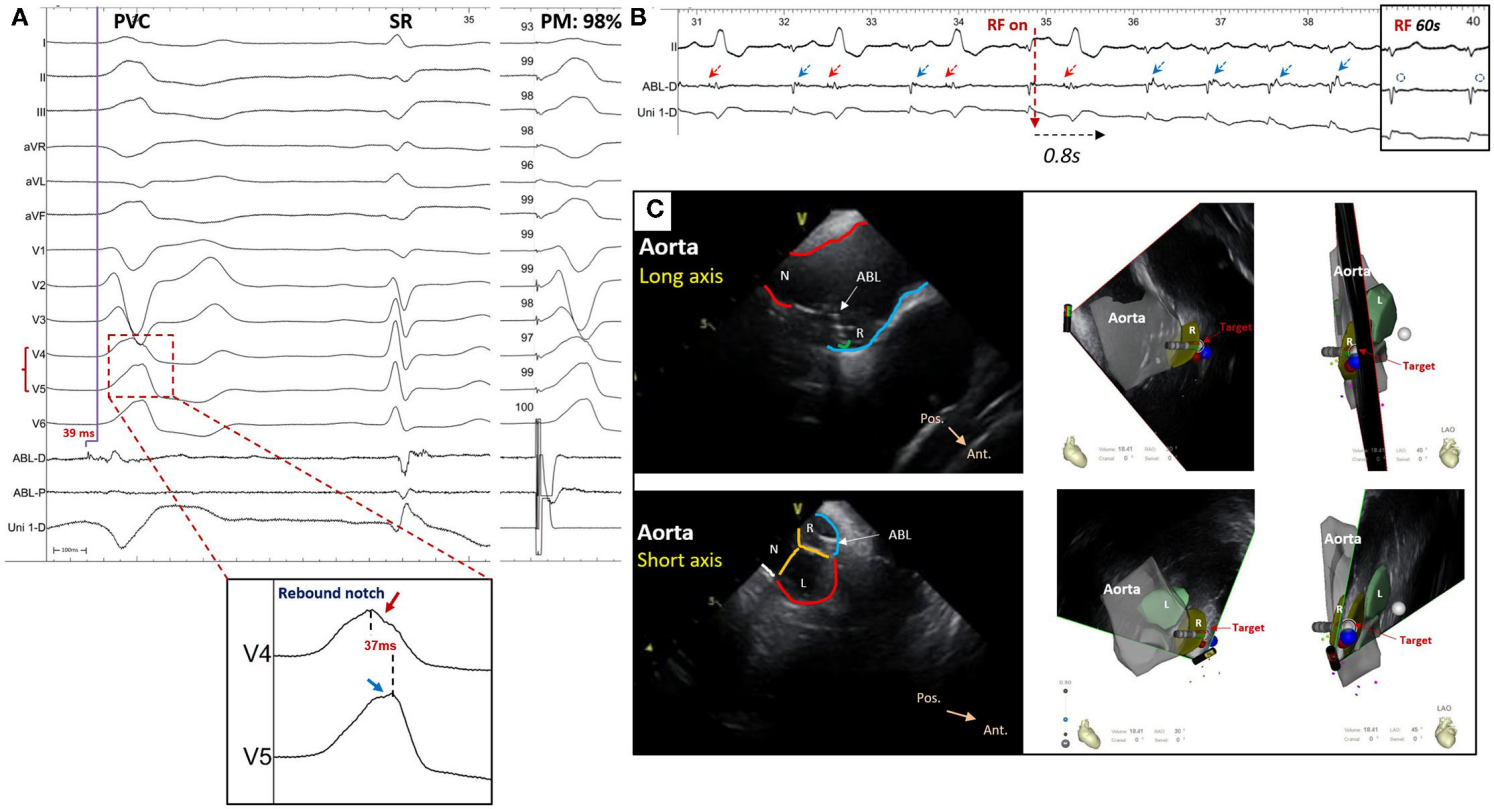
A patient who suffered from premature ventricular contraction (PVC) with left bundle branch block morphology and inferior axis deviation ablated in the right-anterior ASV. **(A)** The ECG of PVC showed a rebound notch on leads V4-V5; the final target revealed a reversal potential (compared with sinus rhythm) that preceded the onset of QRS for 39 ms during PVC and the pace mapping showed a similarity of 98%. **(B)** The ventricular bigeminy rhythm disappeared immediately after radiofrequency (RF) initiation and the late potential (blue arrow) during sinus rhythm was eliminated after 60 s of RF. **(C)** The ablated target was located at the right-anterior ASV through intracardiac echocardiography. ABL-D/P, distal or proximal bipolar electrogram; Uni-D, distal unipolar electrogram; SR, sinus rhythm; PM, pace mapping; ABL, ablation catheter; ASV, aortic sinus of Valsalva; N, non-coronary ASV; L, left ASV; R, right ASV; Pos., posterior; Ant., anterior.

A rebound notch in V3-V4 or V4-V5 (Figure 5) was observed in 91% (10/11) of VAs from the right-anterior ASV, while it was less likely to appear in other subregions (6/40, 15%). Among VAs ablated in the right and left ASV, a rebound notch in V3-V4 or V4-V5 had a sensitivity of 90.9%, a specificity of 85.0%, a positive predictive value (PPV) of 62.5%, and a negative predictive value (NPV) of 97.1% to predict VAs ablated in the right-anterior ASV (Figure 2). Meanwhile, the peak R-wave deflection interval difference between V4-V3 and V5-V4 during VAs was significant between the right-anterior/lateral ASV and other subregions (41 ± 19 vs. 13 ± 17, *p* < 0.01); a cutoff value of 32 ms (area under the curve, 0.82; CI, 0.67–0.98; *p* < 0.01) predicted an ablated target in the right-anterior/lateral ASV with a sensitivity of 83.0% and a specificity of 87.0% (Figure 3).

A rebound notch in V2-V3 during VAs was more common in the left-lateral ASV (6/12; 50 vs. 5%, *p* < 0.05). Among VAs ablated in the right and left ASV, a rebound notch in V2-V3 had a sensitivity of 50.0%, a specificity of 94.9%, a PPV of 75.0%, and a NPV of 86.0% to predict VAs ablated in the left-lateral ASV (Figure 2). Meanwhile, the peak R-wave deflection interval difference between V3 and V2 during VAs seemed greater in the left-lateral ASV, but showed no significance when compared with other subregions (19 ± 16 vs. 12 ± 11, *p* > 0.05). A cutoff value of 31 ms had a sensitivity of 33.3% and a specificity of 92.3% to predict VAs ablated in the left-lateral ASV.

The details of different peak R-wave deflection intervals and precordial rebound notches are given in Supplementary Table 2.

## Discussion

### Major Findings

This study revealed the distribution and electrocardiographic/electrophysiological characteristics of VAs ablated in different subregions of the ASVs. The main findings are as follows:

1. Ventricular arrhythmias from the ASVs are mainly distributed in the anterior and the left lateral portions.
2. Among the 3 kinds of VAs targeting bipolar electrograms in the ASVs, the fractionated potential was in the majority, but its percentage decreased gradually from the right-lateral to the left-lateral ASV.
3. Precordial electrocardiographic rebound notch is a simple and distinct ECG pattern that has a high predictive accuracy for identifying VA origins in the right-anterior ASV or the left-lateral ASV.

### Correlation Between Anatomy and Electrophysiology of the ASV-VAs

As a part of the aortic root, the ASVs are located in the center of the heart, surrounded by the RVOT, the left ventricle, the right atrium, and the left atrium. As for the relative anatomic location, the right ASV forms the right anterior portion of the ASVs and abuts the RVOT, resulting in an electrogram that shows a large ventricular potential and a small (or absent) atrial potential; the left ASV located higher and lateral, which is stuck in the middle of the RVOT and the left atrium and usually shows a local ventricular and atrial electrogram; the non-coronary ASV, which is the lowest and most posterior part, is close to the interatrial septum and shows a large atrial potential (9). Meanwhile, histological study (10) found that ventricular musculature mainly extends into the anterior portion of the ASVs, while the non-coronary ASV, the right-lateral ASV, and the left-lateral ASV are embraced by the fibrous skeleton (11). In keeping with such anatomic substrate, the targets of the ASV-VAs in this study were mainly distributed in the anterior portion of the ASVs, while only a quarter (13/51) of the VA targets was located in the lateral ASVs. However, according to the distributional difference (left-lateral ASV, 13/51 vs. right-lateral ASV, 1/51), the left-lateral ASV should receive more attention than the right-lateral ASV during mapping and ablation, probably due to the anatomic proximity between the left-lateral ASV and the aortomitral continuity [another arrhythmogenic region (12)]. Similarly, Yang et al. (3) reported a case series about the ablation of VAs with predominant monophasic “R” waves in precordial leads from the left ASV. They found that those with small “s” waves in ECG-V2 and a single “V” electrogram in the target were located more inferior and lateral in the left ASV. This anatomic distribution (left-lateral ASV) and these target potential characteristics (lower amplitude and less fractionation) are similar to those in this study, which is why VA targets were more common in the left-lateral ASV than in the right-lateral ASV.

### Electrocardiogram Characteristics of the ASV-VAs

Due to the distinct posterior-anterior and blurry left-right anatomic relationship between the pulmonary root and the aortic root, ECG differentiation of left- and right-sided outflow tract VAs is mainly based on the precordial lead (13). Many ECG signs and prediction algorithms, such as an R-wave duration index ≥ 50% and R/S-wave amplitude index ≥ 30% in V1 and/or V2 (1), a V2 transition ratio ≥ 0.60, a transitional zone index < 0 (7), and an R-wave deflection interval of modified precordial lead (8), can predict an origin of the left ventricular outflow tract VAs. Within the ASVs, different sinuses have different VA ECG characteristics. The right ASV is located inferior, anterior, and to the right of the left ASV, which usually presents as a lower amplitude in the inferior lead, a greater positive portion in lead I, and a later transition in the precordial lead (V2-V3) during VAs recorded in ECG. In contrast to VAs from the right ASV, VAs from the left ASV usually show a larger amplitude in the inferior lead, a greater negative portion in lead I, and an early transition in the precordial lead (before V2) (2). Moreover, some VAs from the left ASV could show predominant monophasic “R” waves in precordial leads with an “s” wave in lead V2 (3). In addition, a “QRS” pattern (5) in leads V1-V3 and/or a “QS” pattern plus a downstroke notch (6) in lead V1 with a precordial transition at lead V3 might suggest a site of VA origin in the left-right commissure. Occasionally, an ECG pattern of narrower QRS duration, “s” wave in lead III, and smaller III/II ratio indicate an ablation in the non-coronary ASV, which might be of a para-Hisian origin. However, none of those studies focused on subregions of the ASV. This study further divided the right and the left ASVs into 6 subregions according to the anatomic marks revealed by ICE or coronary angiography. In addition to the typical ECG characteristics of the ASV-VAs, such as a positive “R” wave in the initiation and termination of lead I being more common in the VAs from the right ASV, we also found that the contiguous precordial electrocardiographic notch patterns could be a novel ECG signature for locating different subregions of the ASV. A left precordial rebound notch (V3-V4/V4-V5) during VAs indicates a right-anterior subregion, while a right precordial rebound notch (V2-V3) during VAs indicates a left-lateral subregion. Above all, this novel precordial ECG sign could provide supplemental evidence for the localization of the ASV-VAs, thus improving the precision and efficiency of mapping in the ASVs.

### Underlying Mechanism of the Precordial Electrocardiographic Rebound Notch

An electrocardiographic notch in VAs might be related to slow or prolonged conduction. Marchlinski et al. first reported that an R wave notch of the inferior lead was more common in the free wall than in the septum during RVOT pacing, which could be explained by the sequential activation of the right ventricle and the left ventricle during pacing from free-wall sites leading to prolonged activation time and causing the electrocardiographic inferior notch (14). Yamada et al. found that VAs originating from the parietal band always presented with a notch in the middle of any QRS in all the cases, which suggested prolonged time conduct from the VA origin to the Purkinje system (15). In addition, the “notch” could be a sign of epicardial VAs. A precordial pseudodelta wave (upstroke notch) ≥ 34 ms has a sensitivity of 83% and a specificity of 95% to predict an epicardial origin (16). Both the upstroke and downstroke notches of the R wave in lead III have a sensitivity of 40.6% and a specificity of 97.5% to predict VAs arising from the distal great cardiac vein (17). Similarly, this study showed that VAs located in the right-anterior ASV, which always show a fractionated potential (slow conduction) in the target bipolar electrogram, are covered by different kinds of precordial rebound notches (V1-V2, 8.3%; V3-V4, 75%; and V4-V5, 16.7%).

A precordial electrocardiographic rebound notch is defined as a downstroke notch on the earlier lead followed by an upstroke notch on the later lead, indicating that the adjacent precordial leads have shorter and longer peak R-wave deflection intervals, respectively. Thus, a precordial electrocardiographic rebound notch is always followed by a larger difference in the peak R-wave deflection interval between the adjacent precordial leads, which may reflect a difference in the local depolarized time. In summary, activations from different subregions of the ASVs are conducted by different vectors, resulting in different patterns of the precordial electrocardiographic rebound notch. Interestingly, we never found a precordial sequential notch (an upstroke notch on the earlier lead followed by a downstroke notch on the later lead) in the study population.

## Limitations

This retrospective study is limited by the small sample size and single center, which prevented us from testing the diagnostic value of precordial electrocardiographic rebound notches in patients with the ASV-VAs in an external validation cohort. Meanwhile, the mechanism of the rebound notch remains speculative and unclear. High-density epicardial and endocardial electroanatomic mapping can reveal the activation pattern of the ASV-VAs and may explain this specific precordial electrocardiographic notch change. Moreover, any target that was confirmed by local activation mapping [28 ms (22–33 ms)] and matching pace mapping could not be definitively recognized as an origin site because we did not perform high-density epicardial and endocardial ventricular outflow tract mapping in all the patients. Thus, we used the verb “originate” prudently. In addition, ICE, considered the gold standard for the location of VAs, was used in only approximately 40% of the population due to the retrospective nature of this study. For the rest of the patients, detailed coronary angiography plus 3-dimensional maps can be an alternative solution. Finally, this study did not enroll patients with VAs originating from the non-coronary ASV because of their low incidence rate and uncertain origin (possibly para-Hisian origin).

## Conclusion

The targets of the ASV-VAs were distributed mainly in the anterior and the left-lateral ASVs. The fractionated potential was dominant in the 3 types of target electrograms, while the percentage of target fractionation gradually decreased from the right-lateral to the left-lateral ASVs. A precordial electrocardiographic rebound notch has high predictive accuracy for the localization of VAs in the right-anterior or left-lateral ASVs.

## Data Availability Statement

The original contributions presented in the study are included in the article/Supplementary Material, further inquiries can be directed to the corresponding author/s.

## Ethics Statement

The studies involving human participants were reviewed and approved by Ethics Committee of Fuwai Hospital. Written informed consent to participate in this study was provided by the participants' legal guardian/next of kin. Written informed consent was obtained from the individual(s) for the publication of any potentially identifiable images or data included in this article.

## Author Contributions

All the authors contributed to the study conception and design. Material preparation, data collection, and analysis were performed by SW, MT, and ZZ. The first draft of the manuscript was written by SW and all the authors commented on previous versions of the manuscript. All the authors read and approved the final version of the manuscript.

## Funding

This work was supported by the National Natural Science Foundation of China (Grant No. 61527811).

## Conflict of Interest

The authors declare that the research was conducted in the absence of any commercial or financial relationships that could be construed as a potential conflict of interest.

## Publisher's Note

All claims expressed in this article are solely those of the authors and do not necessarily represent those of their affiliated organizations, or those of the publisher, the editors and the reviewers. Any product that may be evaluated in this article, or claim that may be made by its manufacturer, is not guaranteed or endorsed by the publisher.

## References

[1] Ouyang F Fotuhi P Ho SY Hebe J Volkmer M Goya M. Repetitive monomorphic ventricular tachycardia originating from the aortic sinus cusp: electrocardiographic characterization for guiding catheter ablation. J Am Coll Cardiol. (2002) 39:500–8. 10.1016/S0735-1097(01)01767-3 11823089

[2] Yamada T McElderry HT Doppalapudi H Murakami Y Yoshida Y Yoshida N. Idiopathic ventricular arrhythmias originating from the aortic root prevalence, electrocardiographic and electrophysiologic characteristics, and results of radiofrequency catheter ablation. J Am Coll Cardiol. (2008) 52:139–47. 10.1016/j.jacc.2008.03.040 18598894

[3] Yang JD Sun Q Guo XG Zhou GB Liu X Luo B. Ablation of ventricular arrhythmias with predominant monophasic “R” waves in precordial leads from the left sinus of Valsalva: Electrocardiographic and electrophysiologic characteristics. J Cardiovasc Electrophysiol. (2019) 30:541–9. 10.1111/jce.13845 30661263

[4] Wang Y Liang Z Wu S Han Z Ren X. Idiopathic ventricular arrhythmias originating from the right coronary sinus: Prevalence, electrocardiographic and electrophysiological characteristics, and catheter ablation. Heart Rhythm. (2018) 15:81–9. 10.1016/j.hrthm.2017.09.008 28917557

[5] Yamada T Yoshida N Murakami Y Okada T Muto M Murohara T. Electrocardiographic characteristics of ventricular arrhythmias originating from the junction of the left and right coronary sinuses of Valsalva in the aorta: the activation pattern as a rationale for the electrocardiographic characteristics. Heart Rhythm. (2008) 5:184–92. 10.1016/j.hrthm.2007.09.029 18242537

[6] Bala R Garcia FC Hutchinson MD Gerstenfeld EP Dhruvakumar S Dixit S. Electrocardiographic and electrophysiologic features of ventricular arrhythmias originating from the right/left coronary cusp commissure. Heart Rhythm. (2010) 7:312–22. 10.1016/j.hrthm.2009.11.017 20097621

[7] Yoshida N Inden Y Uchikawa T Kamiya H Kitamura K Shimano M. Novel transitional zone index allows more accurate differentiation between idiopathic right ventricular outflow tract and aortic sinus cusp ventricular arrhythmias. Heart Rhythm. (2011) 8:349–56. 10.1016/j.hrthm.2010.11.023 21078412

[8] Anderson RD Kumar S Binny S Prabhu M Al-Kaisey A Parameswaran R. Modified precordial lead R-wave deflection interval predicts left- and right-sided idiopathic outflow tract ventricular arrhythmias. JACC Clin Electrophysiol. (2020) 6:1405–19. 10.1016/j.jacep.2020.07.011 33121670

[9] Sasaki T Hachiya H Hirao K Higuchi K Hayashi T Furukawa T. Utility of distinctive local electrogram pattern and aortographic anatomical position in catheter manipulation at coronary cusps. J Cardiovasc Electrophysiol. (2011) 22:521–9. 10.1111/j.1540-8167.2010.01957.x 21091969

[10] Hasdemir C Aktas S Govsa F Aktas EO Kocak A Bozkaya YT. Demonstration of ventricular myocardial extensions into the pulmonary artery and aorta beyond the ventriculo-arterial junction. Pacing Clin Electrophysiol PACE. (2007) 30:534–9. 10.1111/j.1540-8159.2007.00704.x 17437578

[11] Saremi F Sánchez-Quintana D Mori S Muresian H Spicer DE Hassani C. Fibrous skeleton of the heart: anatomic overview and evaluation of pathologic conditions with CT and MR imaging. Radiographics. (2017) 37:1330–51. 10.1148/rg.2017170004 28820653

[12] Chen J Hoff PI Rossvoll O De Bortoli A Solheim E Sun L. Ventricular arrhythmias originating from the aortomitral continuity: an uncommon variant of left ventricular outflow tract tachycardia. Europace. (2012) 14:388–95. 10.1093/europace/eur318 21979993

[13] Anderson RD Kumar S Parameswaran R Wong G Voskoboinik A Sugumar H. Differentiating right- and left-sided outflow tract ventricular arrhythmias. Circul Arrhythmia Electrophysiol. (2019) 12:e007392. 10.1161/CIRCEP.119.007392 31159581

[14] Dixit S Gerstenfeld EP Callans DJ Marchlinski FE. Electrocardiographic patterns of superior right ventricular outflow tract tachycardias: distinguishing septal and free-wall sites of origin. J Cardiovasc Electrophysiol. (2003) 14:1–7. 10.1046/j.1540-8167.2003.02404.x 12625602

[15] Yamada T Yoshida N Itoh T Litovsky SH Doppalapudi H McElderry HT. Idiopathic ventricular arrhythmias originating from the parietal band: electrocardiographic and electrophysiological characteristics and outcome of catheter ablation. Circul Arrhythmia Electrophysiol. (2017) 10:e005099. 10.1161/CIRCEP.117.005099 28794085

[16] Berruezo A Mont L Nava S Chueca E Bartholomay E Brugada J. Electrocardiographic recognition of the epicardial origin of ventricular tachycardias. Circulation. (2004) 109:1842–7. 10.1161/01.CIR.0000125525.04081.4B 15078793

[17] Lin YN Xu J Pan YQ Zheng C Lin JX Li J. An electrocardiographic sign of idiopathic ventricular tachycardia ablatable from the distal great cardiac vein. Heart rhythm. (2020) 17:905–14. 10.1016/j.hrthm.2020.01.027 32028047

