# Supplementary material for: Idiopathic Ventricular Arrhythmias Ablated in Different Subregions of the Aortic Sinuses of Valsalva: Anatomical Distribution, Precordial Electrocardiographic Notch Patterns, and Bipolar Electrographic Characteristics
Source: Front Cardiovasc Med. 2021 Dec 20;8:778866. doi: 10.3389/fcvm.2021.778866 (PMC8720962; doi:10.3389/fcvm.2021.778866)
Supplement: Supplementary file 1 [file Table_1.DOCX]

**Supplementary Table S1** Proportions of target electrogram in different subregions.

| **Target electrogram** | **R-Lat.** | **R-Ant.** | **R-Comm.** | **L-Comm.** | **L-Ant.** | **L-Lat.** | **Sum** |
| --- | --- | --- | --- | --- | --- | --- | --- |
| Single potential | 0 | 0 | 3 | 0 | 0 | 3 | 6 |
| Pre-potential | 0 | 0 | 0 | 2 | 2 | 2 | 6 |
| Fractionated potential | **1(100%)** | **11(100%)** | **10 (77%)** | **7 (78%)** | **3 (60%)** | **7 (58%)** | **39 (76%)** |
| Sum | 1 | 11 | 13 | 9 | 5 | 12 | 51 |

ASV**=** aortic sinus of Valsalva; R-Lat.=right-lateral ASV; R-Ant.=right-anterior ASV; R-Comm.=right side adjacent to the left-right commissure; L-Comm.=left side adjacent to the left-right commissure; L-Ant.=left-anterior ASV; L-Lat.=left-lateral ASV.
