# Supplementary material for: Idiopathic Ventricular Arrhythmias Ablated in Different Subregions of the Aortic Sinuses of Valsalva: Anatomical Distribution, Precordial Electrocardiographic Notch Patterns, and Bipolar Electrographic Characteristics
Source: Front Cardiovasc Med. 2021 Dec 20;8:778866. doi: 10.3389/fcvm.2021.778866 (PMC8720962; doi:10.3389/fcvm.2021.778866)
Supplement: Supplementary file 2 [file Table_2.DOCX]

**Supplementary Table S2** Peak R-wave defection interval and rebound notch of precordial leads.

|  | **R-Lat.**  **(n=1)** | **R-Ant.**  **(n=11)** | **R-Comm.**  **(n=13)** | **L-Comm.**  **(n=9)** | **L-Ant.**  **(n=5)** | **L-Lat.**  **(n=12)** | **P value** |
| --- | --- | --- | --- | --- | --- | --- | --- |
| **QRS duration, ms** | 124 | 150±12 | 142±16 | 147±17 | 153±28 | 146±11 | 0.538 |
| **Peak R-wave defection interval, ms** |  |  |  |  |  |  |  |
| V1 | 54 | 50±20 | 65±26 | 61±18 | 78±37 | 67±16 | 0.267 |
| V2 | 46 | 58±22 | 61±23 | 61±11 | 77±35 | 66±19 | 0.557 |
| V3 | 46 | 66±18 | 77±21 | 78±19 | 84±31 | 85±13 | 0.127 |
| V4 | 87 | 98±26 | 85±19 | 91±19 | 99±19 | 91±13 | 0.605 |
| V5 | 87 | **110±14^*^** | 92±17 | 98±16 | 98±16 | 91±13 | **0.044** |
| V6 | 87 | **111±13^*^** | 92±16 | 98±16 | 99±16 | 88±11 | **0.011** |
| Difference: V3-V2 | 0 | 8±9 | 16±10 | 17±15 | 7±12 | 19±16 | 0.184 |
| Difference: V4-V3 | 41 | **32±24^*^** | 8±9 | 13±22 | 15±23 | 6±8 | **0.006** |
| Difference: V5-V4 | 0 | 12±22 | 7±16 | 8±21 | -1±3 | 0±4 | 0.450 |
| Difference: max {V5-4, V4-3} | 41 | **41±20^*^** | 15±15 | 20±27 | 15±23 | 6±7 | **0.001** |
| **Precordial rebound notch, n(%)** |  |  |  |  |  |  |  |
| None | 0 | 0 | 11 (84) | 5 (56) | 4 (80) | 6 (50) | **-** |
| V1-2 | 0 | 1 (9) | 0 | 0 | 0 | 0 | **-** |
| V2-3 | 0 | 0 | 1 (8) | 1 (11) | 0 | **6 (50)** | **-** |
| V3-4 | 1 (100) | **8 (73)** | 0 | 2 (22) | 1 (20) | 0 | **-** |
| V4-5 | 0 | **2 (18)** | 1 (8) | 1 (11) | 0 | 0 | **-** |

ASV**=**aortic sinus of Valsalva; R-Lat.=right-lateral ASV; R-Ant.=right-anterior ASV; R-Comm.=right side adjacent to the left-right commissure; L-Comm.=left side adjacent to the left-right commissure; L-Ant.=left-anterior ASV; L-Lat.=left-lateral ASV.
